# Complete Genome Sequence of Collinsella aerofaciens JCM 10188^T^

**DOI:** 10.1128/MRA.00134-20

**Published:** 2020-04-16

**Authors:** Dieter M. Tourlousse, Mitsuo Sakamoto, Takamasa Miura, Koji Narita, Akiko Ohashi, Yoshihito Uchino, Atsushi Yamazoe, Keishi Kameyama, Jun Terauchi, Moriya Ohkuma, Hiroko Kawasaki, Yuji Sekiguchi

**Affiliations:** a Biomedical Research Institute, National Institute of Advanced Industrial Science and Technology (AIST), Tsukuba, Ibaraki, Japan; b Microbe Division/Japan Collection of Microorganisms, RIKEN BioResource Research Center, Tsukuba, Ibaraki, Japan; c Biological Resource Center, National Institute of Technology and Evaluation (NITE), Kisarazu, Chiba, Japan; d Japan Microbiome Consortium (JMBC), Osaka, Osaka, Japan; Loyola University Chicago

## Abstract

We report a complete genome sequence of Collinsella aerofaciens JCM 10188^T^ (=VPI 1003^T^). The genome consists of a circular chromosome (2,428,218 bp with 60.6% G+C content) and two extrachromosomal elements. The genome was predicted to contain 5 sets of rRNA genes, 58 tRNA genes, and 2,079 protein-encoding sequences.

## ANNOUNCEMENT

Members of the genus *Collinsella* represent an important group of bacteria in the human gut. *Collinsella* spp. are considered pathobionts, being associated with diseases such as irritable bowel syndrome (1), psoriatic arthritis (2), nonalcoholic steatohepatitis (3), and symptomatic atherosclerosis (4). Elevated abundance of *Collinsella* was also correlated with rheumatoid arthritis (5), with Collinsella aerofaciens being experimentally demonstrated to increase gut permeability and augmented arthritis severity in mice. To allow better understanding of the bacterium's role in the gut, this study provides a complete genome sequence of *C. aerofaciens* JCM 10188^T^ (=VPI 1003^T^), the authentic type strain of this species (6).

Cells were obtained from the Japan Collection of Microorganisms and cultured under an N_2_ atmosphere in modified Gifu anaerobic medium (GAM) broth with 1% glucose. DNA was purified using the EZ1 DNA tissue kit (Qiagen) from cell lysates prepared by bead beating and lysozyme/proteinase K treatment. Short-read libraries were prepared with the TruSeq Nano DNA kit and sequenced on a MiSeq instrument (2 × 251-bp reads) at a coverage of ∼320×. Long-read libraries were constructed with the ligation sequencing kit (SQK-LSK109; Oxford Nanopore Technologies [ONT]) and native barcoding expansion pack (EXP-NBD104; ONT); sequencing was performed on an R9.4.1 flow cell (FLO-MIN106) using the ONT MinION device. All software for read processing and assembly was run with default settings unless indicated otherwise. Base calling was performed with Guppy v3.1.5 (ONT) in high-accuracy mode with concurrent library demultiplexing and barcode trimming. To extract high-quality reads, ONT reads were filtered using NanoFilt v2.5.0 (7) (length, >1,000 bp; quality, ≥9) and then compared to the Illumina reads and quality controlled using Trimmomatic v0.38 (8) with Filtlong v0.2.0 (https://github.com/rrwick/Filtlong); the poorest 10% of read bases were discarded. A total of 68,855 ONT reads (*N*_50_, 6,088 bp; ∼140× coverage) were used to generate a long-read assembly using Flye v2.5 (9). Hybrid assembly was performed with Unicycler v0.4.7 (10) using the Flye assembly and 3,478,246 Illumina reads as inputs. Annotation was performed with the NCBI Prokaryotic Genome Annotation Pipeline v4.11 (11).

The genome of *C. aerofaciens* JCM 10188^T^ contains a circular chromosome of 2,428,218 bp with a G+C content of 60.6%. The assembly also contained two extrachromosomal elements of 23,044 bp (57.3% G+C content) and 4,066 bp (59.3% G+C content), identified as circular by Flye/Unicycler. A BLAST search against NCBI’s nucleotide database indicated that the 23-kbp element had similarity (91% identity and 64% query coverage) to an unidentified plasmid sequence from human feces (GenBank accession number CP021588), whereas the top hit of the 4-kbp element was to a plasmid sequence previously reported in the rat gut metamobilome (GenBank accession number LN852765). Further analyses would, however, be needed to substantiate that both sequences represent genuine extrachromosomal elements and to resolve their function. As a whole, the genome was predicted to contain 5 rRNA operons, 58 tRNA genes, and 2,079 protein-coding sequences.

### Data availability.

The genome sequence has been deposited in DDBJ/EMBL/GenBank under the accession numbers CP048433, CP048434, and CP048435. The raw ONT and Illumina sequencing reads are available in the Sequence Read Archive (SRA) under the accession numbers SRR10968455 and SRR10968458, respectively.
